# Implementing Screening for Neonatal Delirium in the Neonatal Intensive Care Unit: A Quality Improvement Initiative

**DOI:** 10.1097/pq9.0000000000000752

**Published:** 2024-10-21

**Authors:** Meghana Karmarkar, Mark Speziale, Willough Jenkins, Danielle Heath, Jane Kang, Julia Suvak, Peggy Grimm, Laurel Moyer

**Affiliations:** From the * Neonatal ICU, Department of Pediatrics, Kaiser Santa Clara, Santa Clara, Calif.; † Department of Pediatrics, University of California San Diego, La Jolla, Calif.; ‡ Rady Children’s Hospital San Diego, San Diego, Calif.; § Division of Child Psychiatry, Department of Psychiatry, University of California San Diego, La Jolla, Calif.; ¶ Division of Neonatology, Mattel Children’s Hospital, UCLA Santa Monica Medical Center, Santa Monica, Calif.

## Abstract

**Introduction:**

Delirium is not commonly diagnosed in neonatal intensive care units and can adversely impact patient outcomes in the ICU setting. Recognition of delirium in the NICU is a necessary first step to address the potential impact on neonatal outcomes.

**Methods:**

We conducted a quality improvement initiative implementing screening for neonatal delirium. We aimed to increase screening in NICU patients from 0% to 85% by March 2022. Interdisciplinary meetings were held with key stakeholders to develop a clinical algorithm. We used standardized tools for delirium screening. Our process measures included weekly nursing compliance with Richmond Agitation Sedation Scale/Cornell Assessment of Pediatric Delirium/ scoring documentation (Fig. 1) and patients referred to psychiatry. Outcome measures included the percentage of patients screened for delirium before discharge. We conducted Plan-Do-Study Act cycles to optimize the screening process in the electronic medical record (EMR). This included creating an order set, documentation flowsheets, and prompts in the EMR for patients.

**Results:**

After initial implementation, we achieved an average weekly screening compliance of 76% (Fig. 1). Inclusion criteria expansion resulted in a downward compliance shift to 59%. Subsequently, the addition of the EMR checklist resulted in a center-line shift to a sustained average weekly screening compliance of 77%. An average of 82% of all eligible NICU patients received delirium screening before discharge (Fig. 2).

**Conclusions:**

Using quality improvement methodology, there was increased screening and recognition of delirium in our NICU. Future research efforts could focus on assessing preventive measures and the impact of neonatal delirium on patient outcomes.

## Introduction

Delirium is defined as fluctuating changes in consciousness and behavior occurring in the setting of a medical illness. Causes of delirium include underlying medical or surgical illness, side effects of some medications, and medication withdrawal. The prevalence of pediatric delirium in PICUs ranges from 4% to 29%.^1,2^ ICU admission exposes a child to painful and stressful events, including multiple invasive procedures. These events are commonly managed by the administration of analgesics or sedatives. Although adequate analgesia and sedation help reduce the stress response and improve clinical outcomes, overexposure to these agents can lead to prolonged ICU stay, longer time on mechanical ventilation, and delirium.^2–5^ Children who were less than 2 years of age and received opioids or benzodiazepines are more likely to experience symptoms consistent with delirium and have longer lengths of stay compared with older patients.^3^ It can be challenging to distinguish between pain, withdrawal, and delirium in critically ill children. In addition, pediatric delirium is often underdiagnosed in young children, as it is difficult to assess symptoms in nonverbal patients and even more so in those with altered developmental trajectories related to their illness. In a pediatric cardiothoracic ICU that instituted a standard evaluation for delirium, the incidence of delirium after cardiothoracic surgery was 49%.^3^ Based on these findings, there is an increased interest in identifying symptoms of delirium in the neonatal population.

The NICU at Rady Children’s Hospital San Diego (RCHSD) is composed of a medically complex population of patients who are hospitalized for extended periods and require prolonged mechanical ventilation and prolonged exposure to multiple opioid and sedation medications. This population is at increased risk for neonatal delirium. In the pediatric population, untreated delirium can lead to poor neurodevelopmental outcomes, increased duration of mechanical ventilation, increased hospital length of stay, decreased parent satisfaction, and increased morbidity and mortality.^4^ However, there is limited information on delirium in neonates and young infants and potential consequences to their neurodevelopment. Early in life, opioids and benzodiazepines can have harmful effects on the developing brain, including modifications of brain functions, behavioral alterations, and cognitive deficits.^4,6–8^ A case series published in 2015 by Groves et al described three infants presenting with signs of delirium.^9^ All three children had complex medical problems and were receiving multiple analgesic and sedative medications. Each infant exhibited agitation unresponsive to increasing doses of these medications. The initiation of preventive measures and pharmacologic treatment allowed the weaning of other medications, highlighting the importance of recognizing delirium in the neonatal population.

Delirium is related to disturbances in neuroendocrine and inflammatory pathways, and an understanding of normal developmental behavior is required to diagnose delirium in the neonate, making this work particularly challenging with complex neonatal medical care. Signs of possible delirium in a neonate include breathing against the ventilator, requiring escalating doses of pain and sedative medications (including opioids and benzodiazepines), loss of previously acquired milestones, and refractory agitation or inconsolability out of the normal developmental range for the infant.^10^ Infants exhibit normal consciousness during the first 6–8 weeks of life. These signs are summarized in the Cornell Assessment of Pediatric Delirium (CAPD) developmental assessment, which outlines appropriate signs of normal consciousness based on age (**Appendix A** http://links.lww.com/PQ9/A585). The CAPD score is a screening tool used in pediatric ICUs designed to assess critically ill children of all ages for delirium.^11^ Although its use in neonatal intensive care units (NICUs) has not been widely reported, it is the only standardized tool available for infants.^12,13^ Current use of the CAPD in the NICU primarily aids in identifying patients that may require a more extensive delirium evaluation, rather than serving as the primary diagnostic tool for diagnosing delirium. The Richmond Agitation and Sedation Scale (RASS) is a screening tool used before the CAPD score to assess the level of consciousness in a patient. The RASS score helps the observer determine whether to proceed with CAPD scoring based on the level of arousability (**Appendix B** http://links.lww.com/PQ9/A586).

Before this project, there was no standardized means of objectively assessing patients for delirium in our NICU. Utilizing QI methodology and through iterative Plan-Do-Study Act cycles, we developed and implemented a standardized screening tool to identify NICU patients at risk for delirium. Our specific aim was to increase delirium screening (RASS/CAPD scores) from a baseline of 0% to 85% in eligible NICU patients by March 2022.

## METHODS

### Setting

Rady Children’s Hospital is an academic, nonprofit, freestanding children’s hospital in San Diego, California. The 64-bed, level IV NICU receives over 800 admissions annually and a diverse population ranging from extremely low birth weight preterm infants to older medically fragile infants up to a year of age. The NICU staff includes a multidisciplinary team of neonatologists, neonatology fellows, advanced practice nurses, registered nurses (RNs), respiratory therapists (RTs), clinical pharmacists, dieticians, social workers, and occupational and physical therapists.

### Planning the Intervention

The multidisciplinary QI team used the Model for Improvement^14^ framework to create a key driver diagram (Fig. 3), establish a clinical algorithm for recognizing and preventing delirium, and supporting multiple tests of change to improve our processes. The multidisciplinary team included neonatologists, bedside NICU nurses, NICU nursing leadership, child psychiatrists, and a data analyst. We initiated multiple interdisciplinary meetings with key stakeholders to develop a clinical algorithm for evaluating neonatal delirium. We used the completion of the RASS and age-adjusted CAPD scores as the objective tool for delirium screening. Nursing champions trained all NICU bedside nurses how to use the RASS/CAPD screening tool either with one-on-one teaching at the bedside or in nursing staff meetings. Our process measures were weekly nursing compliance with RASS/CAPD scoring documentation in the nursing flowsheets and the successful referral of eligible NICU patients to psychiatry. Outcome measures focused on the percentage of patients with any screening for neonatal delirium before discharge. The team agreed that monitoring the overall volume and appropriateness of psychiatry referrals for delirium consultations would be a balancing measure.

### Interventions

The first step was to develop a clinical algorithm designed to evaluate neonatal delirium. Our delirium screening algorithm is an adaptation of the delirium clinical algorithm by Connecticut Children’s Medical Center. The algorithm was also adapted from the clinical algorithm proposed in Silver et al.’s article on delirium in the pediatric inpatient setting.^12^ We modified the algorithm specifically for our NICU population (**Appendix C** http://links.lww.com/PQ9/A587). The algorithm outlined inclusion criteria for screening, scoring, and evaluation for delirium and agitation prevention. The nursing team integrated several agitation prevention measures into the clinical algorithm implemented for all NICU patients. Preventive measures included: limiting stimulation, minimal handling, observing bundled care times, optimizing lighting conditions in the room, nonnutritive sucking, appropriate use of swaddlers/swings, and decreasing ambient noise.

Next, the team determined the inclusion criteria for screening. The initial inclusion criteria were defined as NICU patients greater than or equal to 38 weeks corrected gestational age (CGA) who were mechanically ventilated for over 7 days and receiving any benzodiazepines or opiates. Strict inclusion criteria were introduced initially to screen only those patients the medical team determined to be at the highest risk for delirium. Subsequent small PDSAs with patients outside of our initial criteria demonstrated that, with our limited scope, we were inadvertently excluding at-risk patients. As a result, we expanded the inclusion criteria to include all infants greater than or equal to 38 weeks CGA in August 2021. Premature infants were excluded from screening because the RASS/CAPD tool only applies to term infants and older. If the RASS score was greater than minus 4, the nurse performed delirium screening once per shift using the CAPD score. If a patient’s CAPD score was consistently greater than 8, an automated order in the electronic medical record (EMR) instructed the bedside nurse to notify the provider. The provider would then discuss care with the bedside nurse to ensure that agitation preventive measures were optimized and to assess any medical issues that may be contributing to elevated scores. Provider assessments included evaluation for sepsis, nutritional assessments, appropriate ventilator management, avoidance of air hunger, correction of electrolyte imbalances, and review of potentially delirium-inducing medications. Patients with consistently elevated CAPD scores despite maximal nonpharmacologic therapy were referred to child psychiatry for further delirium assessment.

We held monthly meetings with the QI team to review compliance data, receive interventions feedback, and develop novel solutions. Feedback and project updates were presented quarterly to nurses and providers at their respective scheduled meetings. The insights gained from this multidisciplinary approach proved invaluable in targeting interventions for development and testing to enhance compliance with the screening process. These additional interventions included modifications to the EMR, which led to improved nursing workflow and greater provider visibility of delirium scores (as well as change in delirium scores). In alignment with stakeholder feedback, we created a designated flowsheet in the EMR for delirium screening in which nurses could record CAPD scores. This allowed scores to be documented in the EMR and tracked over time. Before these modifications, CAPD scores were documented in free text in a clinical note, which made tracking difficult. We also integrated the electronic flowsheet into the nurse’s required documentation. At the end of each shift, the nurse would receive a checklist alert to remind them to complete and document the CAPD score for their patient. In addition, creating this flowsheet allowed CAPD scores to be more readily accessible to the provider, with RASS/CAPD score data included in pre-rounding summaries for the provider to review. We also modified the provider’s daily progress note template to include their patient’s RASS/CAPD scores from the previous 24 hours. These additional interventions improved nursing workflow and increased provider awareness of delirium scores.

### Analysis

Measures were analyzed using statistical process control displayed on P-charts. The analysis of these measures adhered to rule-based conventions for special cause variation, as defined by Provost and Murray.^15,16^

### Ethical Considerations

Upon discussion with the University of California San Diego Health Human Research Protections Program (HRPP) staff, this QI project was deemed nonhuman subjects research and, therefore, exempt from institutional review board (IRB) review.

## RESULTS

**Fig. 1.**
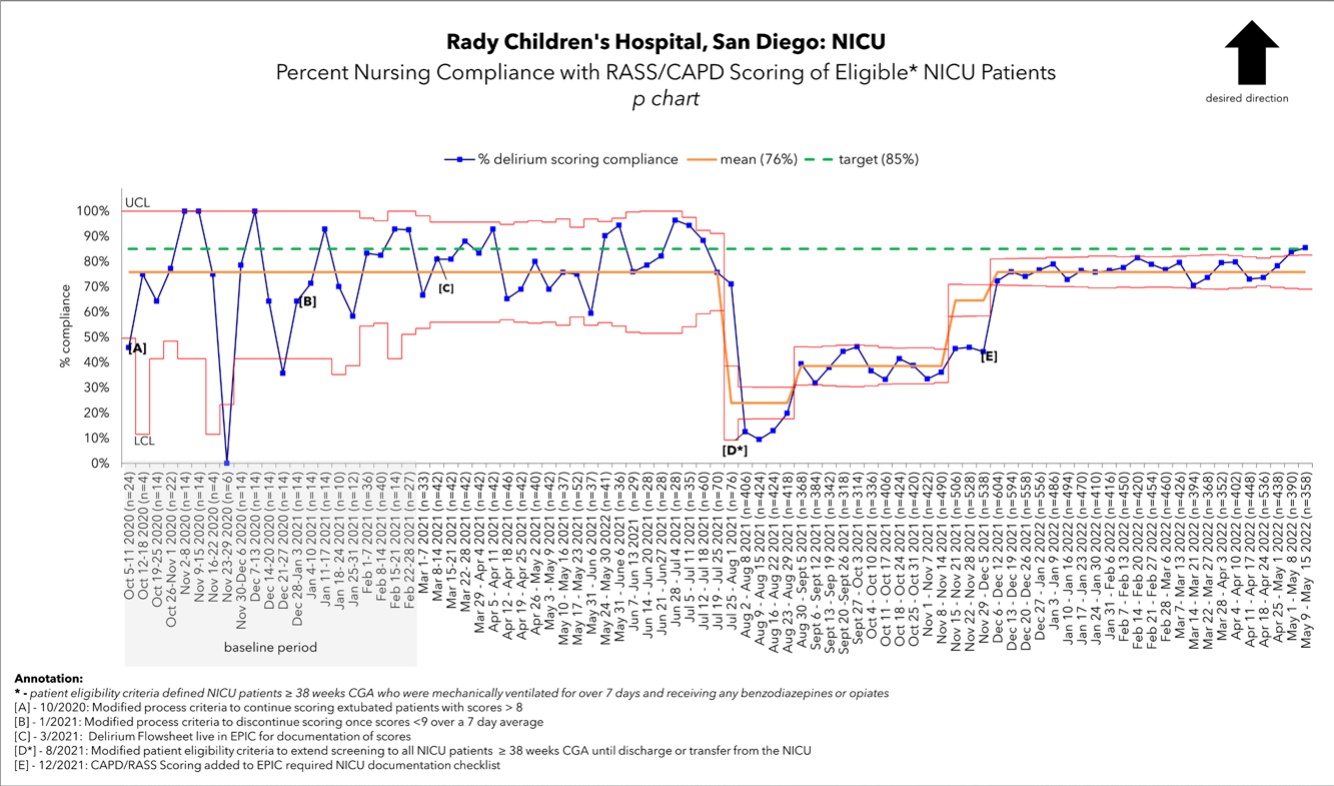
Percent weekly nursing compliance with RASS/CAPD scoring of eligible NICU patients.

**Fig. 2.**
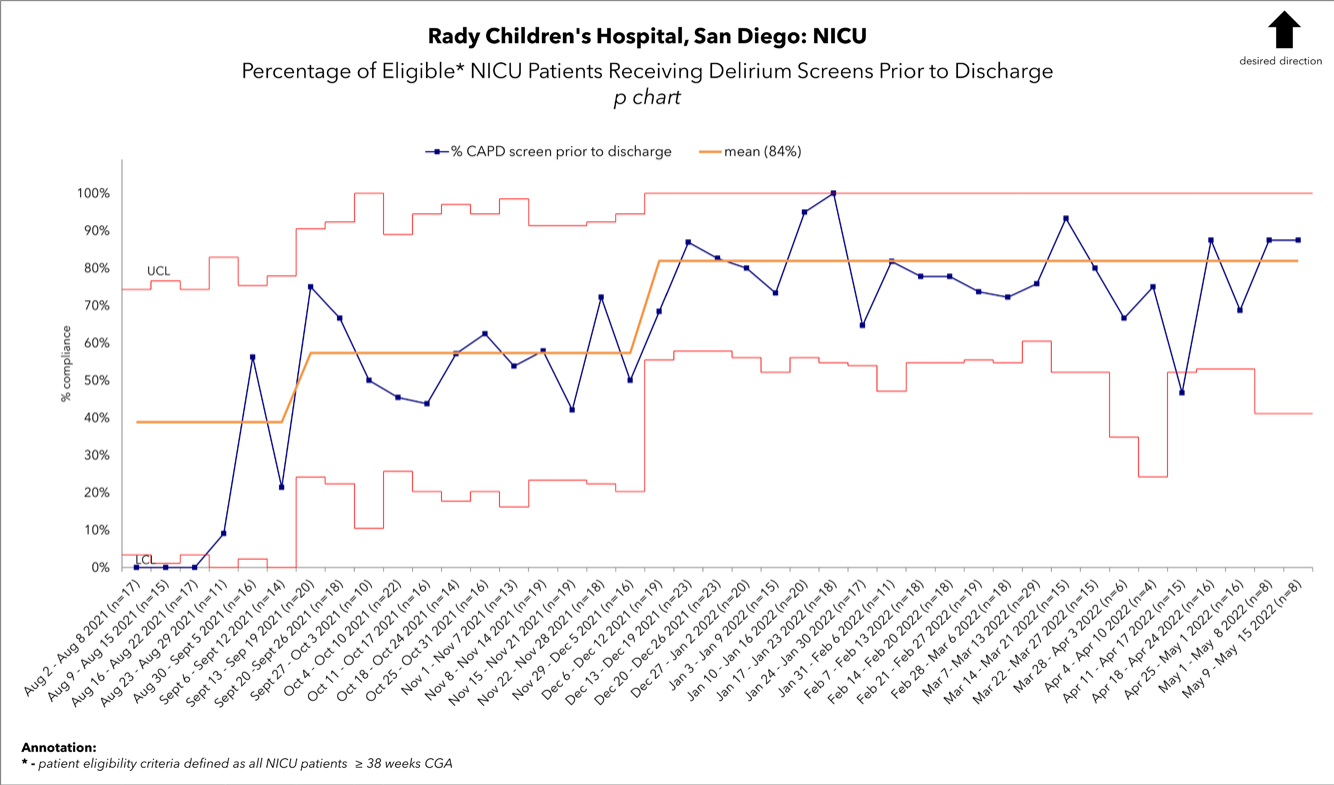
Percentage of eligible NICU patients who received a delirium screen before discharge. -

Implementation of delirium screening and data collection commenced in October 2020. Initially, screening was limited to patients at the highest risk for neonatal delirium.. During the six-month period from October 2020 through February 2021, the data indicated a weekly compliance rate of 76% (Fig. 1). In March 2021, introducing the delirium flowsheet into the EMR for CAPD score documentation and implementing an electronic order set revealed a significant improvement in screening compliance. This improvement was highlighted by the average compliance rate reaching 88%, exceeding the initial goal of 85%. Expansion of the inclusion criteria in August 2021 to include all term infants (greater or equal to 38 weeks CGA) admitted to the NICU resulted in a sharp decline in compliance (Fig. 1). However, integrating the required documentation checklist in the EMR in December 2021 resulted in another positive center-line shift in the data. This increased the average weekly screening compliance to 77%. Because of these efforts, 82% of eligible patients received a delirium screen before discharge (Fig. 2).

**Fig. 3.**
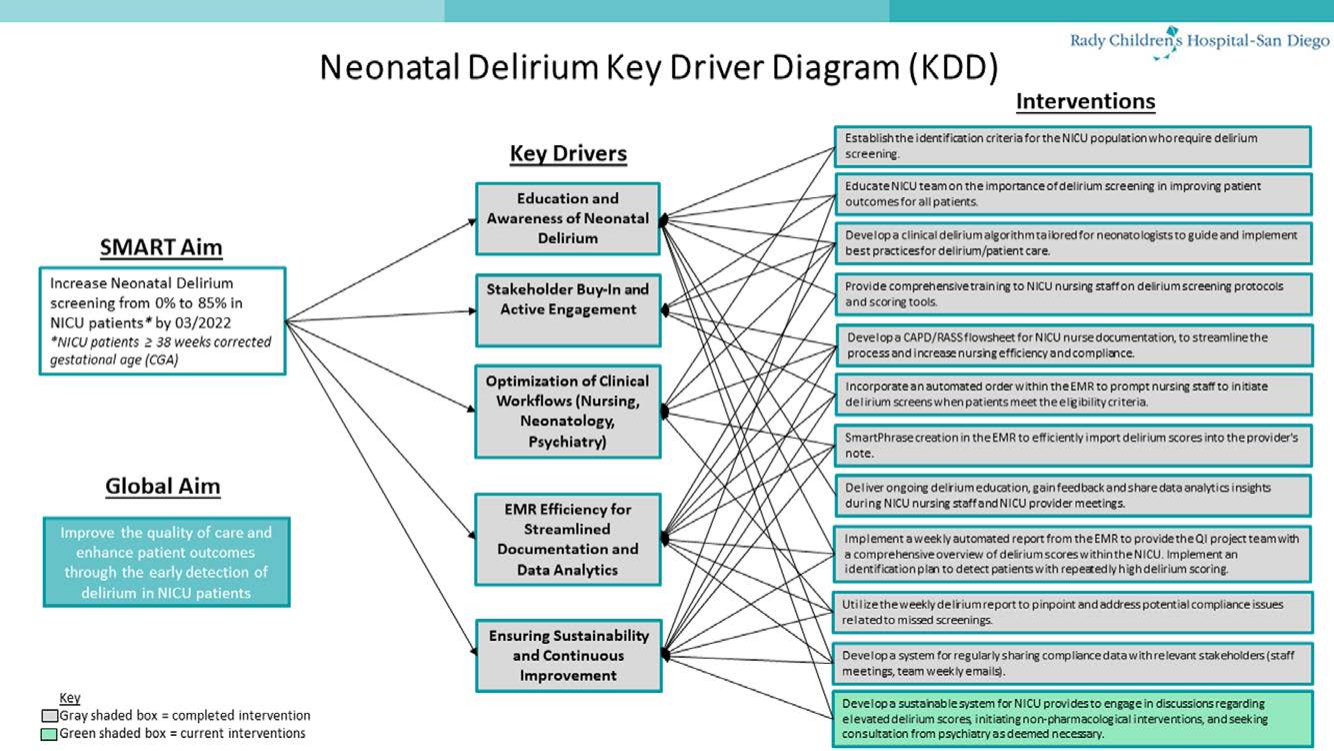
Neonatal Delirium Key Driver Diagram.

As delirium screening became an established routine in our unit, an increased number of patients were identified as high risk for delirium primarily based on consistently high CAPD scores. These patients underwent evaluation by child psychiatry to determine whether their symptoms were suggestive of delirium versus other physiological processes.

## DISCUSSION

In this QI initiative, we demonstrated the feasibility of screening for delirium in the NICU. Consistent with previous case studies, our patients were medically complex and had many symptoms that overlapped with clinical compromise. However, we successfully created a sustainable neonatal delirium screening process by implementing a standardized scoring system, utilizing the EMR, and consultations with our child psychiatry team. By using objective criteria, specifically the CAPD assessment tool, we monitored scores and tracked the progression or regression of symptoms via a shared language. This project demonstrates the value of having an interdisciplinary QI effort aimed at implementing a screening program for delirium in the neonatal population.

Although there have been limited studies documenting pediatric ICU delirium and their experience, this is the first QI project that we are aware of specifically addressing the screening for neonatal delirium in a NICU. We recognize that this work is not transferable to most NICUs where patient populations are predominantly premature infants. Units taking care of more medically complex neonatal patients may benefit from the strategies we found to be successful.

We encountered several challenges during the implementation of this new process. As with any new process for a subset of patients, it was difficult for our bedside nurses to reliably complete the RASS/CAPD screening, as the screening was not previously part of their workflow, and only a small number of patients were receiving delirium screening. In addition, there was no process to escalate concerns to providers regarding elevated scores unless notified by a member of the QI team, which was not a reliable process. To overcome these issues, we used the EMR to implement interventions to create a more sustainable and reliable process.

Upon expanding the criteria to include all infants greater than 38 weeks CGA, there was a significant decrease in screening compliance. However, the initial limited screening highlighted the difficulties of only screening a subpopulation of the unit for an outcome and the barriers to screening and the hazards associated with missing other patients at risk. The expanded inclusion criteria and modifications to the EMR allowed delirium screening to become integrated into the bedside nursing workflow, resulting in an upward trend in compliance. Screening low-risk patients and assessing the criteria producing low score assignments, increased the perceived validity of the screening process and increased motivation for delirium screening. For example, a term infant admitted for a lower acuity condition (such as rule-out sepsis, hyperbilirubinemia, etc.) with consistently low RASS/CAPD scores reinforced the legitimacy of the screening process. With our standardized process and increased screening compliance, providers became increasingly aware of when their patients had elevated scores. Consequently, they would consult child psychiatry more readily to be involved in clinical decision-making.

This QI initiative required bedside nurses to increase their workload by documenting delirium screening on their shift, highlighting the importance of the interdisciplinary team. It was crucial to receive nursing support and feedback for this project and have all team members understand the importance of delirium screening in our patient population.^5^ By creating aids in the medical record to increase the efficiencies of these processes and engagement of the psychiatry service, the staff could appreciate the value of the additional documentation for their patients. We took deliberate steps to emphasize the immense value of documentation for every multidisciplinary team member. Through meaningful discussions, we connected their work to specific patient care examples, highlighting their pivotal role. Additionally, incorporating data into conversations about patient care demonstrated not only the impact of the screening process on care but also contributed to motivating continued commitment to delirium screening.

The collaboration with child psychiatry has increased awareness of delirium as a diagnosis in the NICU. Providers are more aware of the polypharmacy that their patients are receiving and assessing the needs for these medications. In addition, nurses focus on bedside agitation prevention measures and actively advocate for their patients when documenting elevated scores.

Our targeted QI initiative has several strengths and limitations. Before the initial inclusion criteria were expanded to include all infants greater than or equal to >38 weeks CGA, our weekly sample of patients undergoing screening was small, making interpretation of compliance difficult. However, expanding the inclusion criteria allowed for increased evaluation of our at-risk patients and improved the reliability of the processes with a larger patient population. Although there remains little evidence for diagnosing and screening for neonatal delirium,^5^ we chose to implement the best available screening tool to identify high-risk patients in the NICU. We focused our improvement effort on the delirium screening process and omitted the inclusion of sedation utilization data and pharmacotherapy as it falls outside the scope of the initial project. This limitation does not allow the reader to draw conclusions regarding associations between observed improvements and factors such as neonatal delirium identification, medication exposures, or clinical outcomes.

Although our current screening compliance stands at 77%, persistent obstacles still demand ongoing efforts to improve compliance. Although nurses have successfully integrated delirium screening into their workflow, ongoing education for delirium scoring among new staff and developing a method to assess interrater reliability must be addressed. Healthcare providers should interpret scores consistently and seek a prompt referral to child psychiatry. When evaluating the overall compliance with delirium screening in the NICU, an average of 82% of patients received at least one initial delirium screen before discharge, indicating most patients do undergo screening. This showcases our commitment to screening and a notable shift in our approach to evaluating neonatal delirium. Future research efforts could be directed to assessing preventive measures in the NICU and the potential impact of neonatal delirium on patient outcomes.

## CONCLUSIONS

Although we focused our project on delirium screening, the methodology and interventions can apply to other units looking to implement a new process. Drafting a clinical algorithm, collaborating with nursing, and optimizing the EMR are all tools that can be beneficial when a unit desires to implement a new process.

Through this QI initiative, we have increased delirium screening in our NICU and awareness of neonatal delirium as a diagnosis. As therapies develop for more complex diseases, NICUs will see an increase in patients who suffer from neonatal delirium, and early recognition will be important to their overall recovery. We suggest that the processes described here can be applied to other NICUs for the early recognition of neonatal delirium and may lead to studies of the development of preventive practices and the impact of neonatal delirium on patient outcomes.

## Supplementary Material


