# Supplementary material for: Implementing Screening for Neonatal Delirium in the Neonatal Intensive Care Unit: A Quality Improvement Initiative
Source: Pediatr Qual Saf. 2024 Oct 21;9(6):e752. doi: 10.1097/pq9.0000000000000752 (PMC11495695; doi:10.1097/pq9.0000000000000752)
Supplement: Supplementary file 1 [file pqs-9-e752-s001.pdf]

	Newborn	4 weeks	6 weeks	8 weeks	28 weeks	1 year	2 years
1. Is the child able to make eye contact with the person caring for him/her?	Looks at faces.	Maintains gaze for short periods of time.<br><br>Eyes follow for 90 degrees.	Maintains gaze.	Eyes follow caregiver or objects crossing his/her center line. Pays attention to object held by tester.	Maintains gaze.<br>Prefers parent.<br>Looks at person talking.	Maintains gaze.<br>Prefers parent.<br>Looks at person talking.	Maintains gaze.<br>Prefers parent.<br>Looks at person talking.
2. Does the child engage in purposeful actions?	Moves head side to side in accordance with neonatal reflex.	Stretches hand out. (OK if this action is somewhat uncoordinated).	Stretches out hand.	Tries to grasp object offered using contrasting side to side movements without resistance.	Stretches out hand smoothly.	Stretches out hand and tries to grasp object. Tries to change position. If able to move, tries to stand.	Stretches out hand and tries to grasp object. Tries to change position. If able to move, tries to stand.
3. Is the child interested in his/her surroundings?	The child is calm and alert.	The child is clearly alert.<br><br>The child turns in the direction of the caregiver's voice.<br><br>The child may turn in the direction of the smell of the caregiver.	The amount of time the child is clearly alert increases<br><br>The child turns in the direction of the caregiver's voice<br><br>The child may turn in the direction of the smell of the caregiver	Nods head up and down, frowns at the sound of a bell, spoken to gently, expression becomes bright and smiles.	Prefers his/her mother over other family members.<br>Becomes used to new objects and can distinguish between objects.	Prefers parents over other family members. Becomes agitated if separated from the preferred caregiver.<br>Is used to a favorite blanket or stuffed animal and is calmed by it.	Prefers parents over other family members. Becomes agitated if separated from the preferred caregiver.<br>Is used to a favorite blanket or stuffed animal and is calmed by it.
4. Does the child communicate his/her needs and wants?	Cries when hungry or uncomfortable.	Cries when hungry or uncomfortable.	Cries when hungry or uncomfortable.	Cries when hungry or uncomfortable.	Vocalizes or uses gestures when needs something (e.g., hungry, uncomfortable, interested in an object or his/her surroundings).	Uses single words and gestures.	Uses 3-4 words and gestures. Indicates when needs to use the toilet.
5. Is the child restless?	Does not maintain a clearly alert state.	Does not maintain a calm state.	Does not maintain a calm state.	Does not maintain a calm state.	Does not maintain a calm state.	Does not maintain a calm state.	Does not maintain a calm state.
6. Is it impossible to console the child?	Cannot be soothed by rocking, singing, feeding, or making comfortable.	Cannot be soothed by rocking, singing, feeding, or making comfortable.	Cannot be soothed by rocking, singing, feeding, or making comfortable.	Cannot be soothed by rocking, singing, feeding, or making comfortable.	Cannot be soothed using normally used methods (e.g., singing, holding, or talking).	Cannot be soothed using normally used methods (e.g., singing, holding, talking, or reading a book).	Cannot be soothed using normally used methods (e.g., singing, holding, talking, or reading a book), but can be calmed down when throwing a temper tantrum.
7. Has the child's level of activity decreased? Has the amount of movement while he/she is awake decreased?	Almost never bends arms and legs, has no strength other than during neonatal reflex.<br><br>(Most of the time the child is sleeping comfortably).	Almost never stretches hands out, kicks, or grasps objects (OK even if actions are somewhat uncoordinated).	Almost never stretches hands out, kicks, or grasps objects (OK even if actions are somewhat uncoordinated).	Almost never grasps objects or moves head and arms purposefully. For example, does not try to push away unpleasant things.	Almost never stretches hands out, grasps objects, moves around on the bed, or pushes away objects.	Almost never plays, gets up, or pulls. Even if he/she moves, almost never crawls or walks around.	Almost never engages in more complex play, gets up, or moves around. Almost never stands, walks, or jumps, even though capable of these actions.
8. Does it take time for the child to react to others?	Does not make sounds. Does not react as expected (e.g., grasping reflex, sucking reflex, or the Moro reflex).	Does not make sound. Does not react as expected (e.g., grasping reflex, sucking reflex, or the Moro reflex).	Does not kick or cry in response to an unpleasant stimulus.	Does not babble, laugh, or gaze in reaction to contact with others.	Does not babble, smile, or laugh during contact with others (may even refuse contact).	Does not obey simple instructions. Even if able to understand, does not obey simple instructions provided in the language that can be understood by the child.	Does not obey simple commands with one to two steps. Even if able to speak, does not obey more complex instructions.
