# Supplementary material for: Implementing Screening for Neonatal Delirium in the Neonatal Intensive Care Unit: A Quality Improvement Initiative
Source: Pediatr Qual Saf. 2024 Oct 21;9(6):e752. doi: 10.1097/pq9.0000000000000752 (PMC11495695; doi:10.1097/pq9.0000000000000752)
Supplement: Supplementary file 2 [file pqs-9-e752-s002.pdf]

Table 1. The Richmond Agitation-Sedation Scale (RASS)

Score	Term	Description	
+4	Combative	Overtly combative, violent, immediate danger to staff	
+3	Very agitated	Pulls or removes tube(s) or catheter(s); aggressive	
+2	Agitated	Frequent nonpurposeful movement, fights ventilator	
+1	Restless	Anxious but movements not aggressive or vigorous	
0	Alert and calm		
−1	Drowsy	Not fully alert, but has sustained awakening (eye opening/eye contact) to voice (>10 seconds)	Verbal stimulation
−2	Light sedation	Briefly awakens with eye contact to voice (<10 seconds)	Verbal stimulation
−3	Moderate sedation	Movement or eye opening to voice (but no eye contact)	Verbal stimulation
−4	Deep sedation	No response to voice, but movement or eye opening to physical stimulation	Physical stimulation
−5	Unarousable	No response to voice or physical stimulation	Physical stimulation

Procedure for RASS Assessment

1. Observe patient
   - Patient is alert, restless, or agitated. — Score 0 to +4
2. If not alert, state patient's name and say to open eyes and look at speaker.
   - Patient awakens with sustained eye opening and eye contact. — Score −1
   - Patient awakens with eye opening and eye contact, but not sustained. — Score −2
   - Patient has any movement in response to voice but no eye contact. — Score −3
3. When no response to verbal stimulation, physically stimulate patient by shaking shoulder and/or rubbing sternum.
   - Patient has any movement to physical stimulation. — Score −4
   - Patient has no response to any stimulation. — Score −5

Adapted with permission.[29]

Please answer the following questions based on your interactions with the patient over the course of your shift:

	Never	Rarely	Sometimes	Often	Always	Score
	4	3	2	1	0	
**1. Does the child make eye contact with the caregiver?**						
**2. Are the child's actions purposeful?**						
**3. Is the child aware of his/her surroundings?**						
**4. Does the child communicate needs and wants?**						
	**Never**	**Rarely**	**Sometimes**	**Often**	**Always**	
	0	1	2	3	4	
**5. Is the child restless?**						
**6. Is the child inconsolable?**						
**7. Is the child underactive—very little movement while awake?**						
**8. Does it take the child a long time to respond to interactions?**						
					**TOTAL**	
