# Supplementary material for: Implementing Screening for Neonatal Delirium in the Neonatal Intensive Care Unit: A Quality Improvement Initiative
Source: Pediatr Qual Saf. 2024 Oct 21;9(6):e752. doi: 10.1097/pq9.0000000000000752 (PMC11495695; doi:10.1097/pq9.0000000000000752)
Supplement: Supplementary file 3 [file pqs-9-e752-s003.pdf]

Rady Children's Hospital-San Diego

# Rady Children's Hospital
# Neonatal Delirium Clinical Algorithm—Prevention

THIS ALGORITHM SERVES AS A GUIDE AND DOES NOT REPLACE CLINICAL JUDGMENT.

**Screening Inclusion Criteria**: all NICU patients > or = 38 weeks CGA

Concurrent implementation of preventive strategies and delirium screening as outlined below

## Preventive Strategies

**Environmental Considerations:**

- Cluster Care, establish daily routines, primary nurses, utilize same room
- Familiar objects in room
- Promote family presence at bedside
- Normalize bodily functions (optimize nutrition, bowel movement)
- Promote regular day/night schedules (maximize sunlight during day, decrease artificial lighting, room temperature)
- Noise pollution prevention (e.g. music therapy)
- Promote mobility (PT/OT, child life therapy)

**Medication Considerations:**

- Assess, prevent and manage pain effectively
- Assess daily sedative and pain medication need and effectiveness, wean as able
- Monitor and prevent withdrawal
- Minimize polypharmacy and delirium-inducing medications as appropriate*

***Deliriogenic Medications:**

- Benzodiazepines
- Narcotics
- Anti-cholinergic
- Ondansetron (Zofran)
- Ranitidine (Zantac)
- Metoclopramide (Reglan)

## Delirium Screening

RN to perform delirium screening using RASS scoring and CAPD (Cornell Assessment of Pediatric Delirium) q12hr (Appendix A) and document in medical record

High clinical suspicion of Delirium? **CAPD > 8**

**No:**

- Continue prevention and ongoing monitoring via CAPD q12hr

**Yes:**

- Notify NICU provider from primary medical team
- If CAPD >8 (over a 7-day average), providers should consult with Child psychiatry (if not consulted already) for recommendations on management
- Provider to initiate a bedside assessment of patient and proceed to **Delirium management pathway**

# Rady Children's Hospital
# Neonatal Delirium Clinical Algorithm—Management

THIS ALGORITHM SERVES AS A GUIDE AND DOES NOT REPLACE CLINICAL JUDGMENT.

### Treat suspected etiology

- Treat suspected etiology per primary and consulting teams, as appropriate

### Physiologic Management

**Workup & Assessment**

- Consider alternate explanations for agitation/restlessness (e.g. neurological injury, pain, air hunger):
- Optimize oxygenation/ventilation
- Correct electrolyte imbalances
- Assess for infection/sepsis
- Evaluate for intracranial process or seizures
- Minimize deliriogenic meds (including benzodiazepines, narcotics, ondansetron, metoclopramide, anticholinergics, ranitidine)
- Optimize pain control with non-pharmacologic strategies
- Monitor and prevent withdrawal
- Assess sedative medication need and effectiveness, wean as able

### Nursing Care

**Monitoring and Safety**

- Vitals per unit policy
- Continue monitoring for delirium via q12 hour CAPD scores (Appendix A)
- Ensure safe handling
- Seizure precautions if necessary
- Reduce or avoid physical restraints
- Engage and educate parents

### Optimize environment

**How to optimize**

- Cluster Care, establish daily routines, primary nurses, utilize same room
- Familiar objects in room
- Promote family presence at bedside
- Normalize bodily functions (optimize nutrition, bowel movement)
- Promote regular day/night schedules (maximize sunlight during day, decrease artificial lighting, room temperature)
- Noise pollution prevention (e.g. music therapy)
- Promote mobility (PT/OT, child life therapy)

### Symptoms improving? (CAPD scores <9 or down trending)

**NO**

- **Consult Child Psychiatry for recommendations on pharmacologic management and determination of length of screening**
- Optimize precedex use for sedation
- Consider melatonin for sleep
- Consider antipsychotic (Seroquel) for agitation
- Continue to optimize environment and medications

**YES**

- Continue to optimize environment specifically as noted above
- Wean antipsychotic/pain/sedation medications, in consultation with psychiatry

# Rady Children's Hospital
# Neonatal Delirium—Appendix A: RASS and CAPD Scoring

THIS ALGORITHM SERVES AS A GUIDE AND DOES NOT REPLACE CLINICAL JUDGMENT.

## Neonatal Delirium Algorithm for Screening

## Step 1

**Eligibility Criteria**

- ⇒ Mechanical ventilation >7 days
- ⇒ ≥38 weeks CGA **AND** Benzodiazepine and/or opioid use
- ⇒ Non-palliative care

## Step 2

At the end of each shift (Note, the following scoring should be based on the bedside nurse's **overall** assessment of the patient **over his/her shift**, not how the patient appears at the time of the assessment)

https://www.mdcalc.com/richmond-agitation-sedation-scale-rass can take you through the above RASS assessment

**Table 1.** The Richmond Agitation-Sedation Scale (RASS)

| Score | Term | Description | |
|---|---|---|---|
| +4 | Combative | Overtly combative, violent, immediate danger to staff | |
| +3 | Very agitated | Pulls or removes tube(s) or catheter(s); aggressive | |
| +2 | Agitated | Frequent nonpurposeful movement, fights ventilator | |
| +1 | Restless | Anxious but movements not aggressive or vigorous | |
| 0 | Alert and calm | | |
| −1 | Drowsy | Not fully alert, but has sustained awakening (eye opening/eye contact) to voice (>10 seconds) | Verbal stimulation |
| −2 | Light sedation | Briefly awakens with eye contact to voice (<10 seconds) | |
| −3 | Moderate sedation | Movement or eye opening to voice (but no eye contact) | |
| −4 | Deep sedation | No response to voice, but movement or eye opening to physical stimulation | Physical stimulation |
| −5 | Unarousable | No response to voice or physical stimulation | |

Procedure for RASS Assessment

1. Observe patient
   - Patient is alert, restless, or agitated. — Score 0 to +4
2. If not alert, state patient's name and say to open eyes and look at speaker.
   - Patient awakens with sustained eye opening and eye contact. — Score −1
   - Patient awakens with eye opening and eye contact, but not sustained. — Score −2
   - Patient has any movement in response to voice but no eye contact. — Score −3
3. When no response to verbal stimulation, physically stimulate patient by shaking shoulder and/or rubbing sternum.
   - Patient has any movement to physical stimulation. — Score −4
   - Patient has no response to any stimulation. — Score −5

## Step 3

If RASS score > or = -4, proceed to Cornell Assessment of Pediatric Delirium (CAPD) Screening. (Note, the following scoring should be based on the bedside nurse's **overall** assessment of the patient **over his/her shift**, not how the patient appears at the time of the assessment)

https://www.mdcalc.com/cornell-assessment-pediatric-delirium-capd can take you through the CAPD assessment based on patient's developmental age or; use developmental tool below to assist in scoring

**Figure 1.** Cornell Assessment of Pediatric Delirium (CAPD) revised

**RASS Score ____**

**Please answer the following questions based on your interactions with the patient over the course of your shift:**

| | Never | Rarely | Sometimes | Often | Always | Score |
|---|---|---|---|---|---|---|
| | 4 | 3 | 2 | 1 | 0 | |
| 1. Does the child make eye contact with the caregiver? | | | | | | |
| 2. Are the child's actions purposeful? | | | | | | |
| 3. Is the child aware of his/her surroundings? | | | | | | |
| 4. Does the child communicate needs and wants? | | | | | | |
| | Never | Rarely | Sometimes | Often | Always | |
| | 0 | 1 | 2 | 3 | 4 | |
| 5. Is the child restless? | | | | | | |
| 6. Is the child inconsolable? | | | | | | |
| 7. Is the child underactive—very little movement while awake? | | | | | | |
| 8. Does it take the child a long time to respond to interactions? | | | | | | |
| | | | | | TOTAL | |

***Developmental Guide for CAPD Scoring on reverse side***

## Step 4

- ☐ If CAPD >8, alert NICU medical provider. Continue to optimize Delirium prevention strategies per pathway and continue qshift RASS assessments and CAPD scoring.
- ☐ If CAPD <9, continue to perform qshift RASS assessments and CAPD scoring until instructed to discontinue by provider.

## Step 5

Documentation of **RASS** and CAPD Scores: Document **RASS** and CAPD score and breakdown of scores qshift through nursing progress note

**Bedside Delirium Prevention Strategies**

- ⇒ Environmental: Maximize sunlight, decrease artificial lighting, room temperature control, minimize room changes
- ⇒ Family presence at bedside and involvement in cares when possible
- ⇒ Facilitate mobilization with developmental-appropriate mobility tools
- ⇒ Familiar objects in room
- ⇒ Cluster care/limited stimulation and handling to facilitate uninterrupted sleep (when possible)
- ⇒ Establish daily routines
- ⇒ Noise contamination reduction

# Rady Children's Hospital
# Neonatal Delirium—Appendix A: RASS and CAPD Scoring

THIS ALGORITHM SERVES AS A GUIDE AND DOES NOT REPLACE CLINICAL JUDGMENT

## Developmental Guide for CAPD Scoring

| | Newborn | 4 weeks | 6 weeks | 8 weeks | 28 weeks | 1 year | 2 years |
|---|---|---|---|---|---|---|---|
| 1. Is the child able to make eye contact with the person caring for him/her? | Looks at faces. | Maintains gaze for short periods of time.<br><br>Eyes follow for 90 degrees. | Maintains gaze. | Eyes follow caregiver or objects crossing his/her center line. Pays attention to object held by tester. | Maintains gaze. Prefers parent. Looks at person talking. | Maintains gaze. Prefers parent. Looks at person talking. | Maintains gaze. Prefers parent. Looks at person talking. |
| 2. Does the child engage in purposeful actions? | Moves head side to side in accordance with neonatal reflex. | Stretches hand out. (OK if this action is somewhat uncoordinated). | Stretches out hand. | Tries to grasp object offered using contrasting side to side movements without resistance. | Stretches out hand smoothly. | Stretches out hand and tries to grasp object. Tries to change position. If able to move, tries to stand. | Stretches out hand and tries to grasp object. Tries to change position. If able to move, tries to stand. |
| 3. Is the child interested in his/her surroundings? | The child is calm and alert. | The child is clearly alert.<br><br>The child turns in the direction of the caregiver's voice.<br><br>The child may turn in the direction of the smell of the caregiver. | The amount of time the child is clearly alert increases<br><br>The child turns in the direction of the caregiver's voice<br><br>The child may turn in the direction of the smell of the caregiver | Nods head up and down, frowns at the sound of a bell, spoken to gently, expression becomes bright and smiles. | Prefers his/her mother over other family members. Becomes used to new objects and can distinguish between objects. | Prefers parents over other family members. Becomes agitated if separated from the preferred caregiver. Is used to a favorite blanket or stuffed animal and is calmed by it. | Prefers parents over other family members. Becomes agitated if separated from the preferred caregiver. Is used to a favorite blanket or stuffed animal and is calmed by it. |
| 4. Does the child communicate his/her needs and wants? | Cries when hungry or uncomfortable. | Cries when hungry or uncomfortable. | Cries when hungry or uncomfortable. | Cries when hungry or uncomfortable. | Vocalizes or uses gestures when needs something (e.g., hungry, uncomfortable, interested in an object or his/her surroundings). | Uses single words and gestures. | Uses 3-4 words and gestures. Indicates when needs to use the toilet. |
| 5. Is the child restless? | Does not maintain a clearly alert state. | Does not maintain a calm state. | Does not maintain a calm state. | Does not maintain a calm state. | Does not maintain a calm state. | Does not maintain a calm state. | Does not maintain a calm state. |
| 6. Is it impossible to console the child? | Cannot be soothed by rocking, singing, feeding, or making comfortable. | Cannot be soothed by rocking, singing, feeding, or making comfortable. | Cannot be soothed by rocking, singing, feeding, or making comfortable. | Cannot be soothed by rocking, singing, feeding, or making comfortable. | Cannot be soothed using normally used methods (e.g., singing, holding, or talking). | Cannot be soothed using normally used methods (e.g., singing, holding, talking, or reading a book). | Cannot be soothed using normally used methods (e.g., singing, holding, talking, or reading a book), but can be calmed down when throwing a temper tantrum. |
| 7. Has the child's level of activity decreased? Has the amount of movement while he/she is awake decreased? | Almost never bends arms and legs, has no strength other than during neonatal reflex.<br><br>(Most of the time the child is sleeping comfortably). | Almost never stretches hands out, kicks, or grasps objects (OK even if actions are somewhat uncoordinated). | Almost never stretches hands out, kicks, or grasps objects (OK even if actions are somewhat uncoordinated). | Almost never grasps objects or moves head and arms purposefully. For example, does not try to push away unpleasant things. | Almost never stretches hands out, grasps objects, moves around on the bed, or pushes away objects. | Almost never plays, gets up, or pulls. Even if he/she moves, almost never crawls or walks around. | Almost never engages in more complex play, gets up, or moves around. Almost never stands, walks, or jumps, even though capable of these actions. |
| 8. Does it take time for the child to react to others? | Does not make sounds. Does not react as expected (e.g., grasping reflex, sucking reflex, or the Moro reflex). | Does not make sound. Does not react as expected (e.g., grasping reflex, sucking reflex, or the Moro reflex). | Does not kick or cry in response to an unpleasant stimulus. | Does not babble, laugh, or gaze in reaction to contact with others. | Does not babble, smile, or laugh during contact with others (may even refuse contact). | Does not obey simple instructions. Even if able to understand, does not obey simple instructions provided in the language that can be understood by the child. | Does not obey simple commands with one to two steps. Even if able to speak, does not obey more complex instructions. |

Adapted from Connecticut Children's Medical Center Delirium Clinical Pathway, 2019.
